# Embodied cognitive flexibility and neuroplasticity following Quadrato Motor Training

**DOI:** 10.3389/fpsyg.2015.01021

**Published:** 2015-07-22

**Authors:** Tal D. Ben-Soussan, Aviva Berkovich-Ohana, Claudia Piervincenzi, Joseph Glicksohn, Filippo Carducci

**Affiliations:** ^1^The Leslie and Susan Gonda (Goldschmied) Multidisciplinary Brain Research Center, Bar-Ilan UniversityRamat Gan, Israel; ^2^Research Institute for Neuroscience, Education and Didactics, Patrizio Paoletti FoundationAssisi, Italy; ^3^Department of Neurobiology, Weizmann Institute of ScienceRehovot, Israel; ^4^Department of Physiology and Pharmacology, Sapienza University of RomeRome, Italy; ^5^Institute for Advanced Biomedical Technologies, Università degli Studi Gabriele D’AnnunzioChieti, Italy; ^6^Department of Criminology, Bar-Ilan UniversityRamat Gan, Israel

**Keywords:** Quadrato Motor Training, creativity, cerebellum, MRI, embodiment

## Abstract

Quadrato Motor Training (QMT) is a whole-body movement contemplative practice aimed at increasing health and well-being. Previous research studying the effect of one QMT session suggested that one of its means for promoting health is by enhancing cognitive flexibility, an important dimension of creativity. Yet, little is known about the effect of a longer QMT practice on creativity, or the relative contribution of the cognitive and motor aspects of the training. Here, we continue this line of research in two inter-related studies, examining the effects of prolonged QMT. In the first, we investigated the effect of 4-weeks of daily QMT on creativity using the Alternate Uses (AUs) Task. In order to determine whether changes in creativity were driven by the cognitive or the motor aspects of the training, we used two control groups: *Verbal Training* (VT, identical cognitive training with verbal response) and *Simple Motor Training* (SMT, similar motor training with reduced choice requirements). Twenty-seven participants were randomly assigned to one of the groups. Following training, cognitive flexibility significantly increased in the QMT group, which was not the case for either the SMT or VT groups. In contrast to one QMT session, ideational fluency was also significantly increased. In the second study, we conducted a pilot longitudinal structural magnetic resonance imaging and diffusion tensor imaging (4-weeks QMT). We report gray matter volume and fractional anisotropy changes, in several regions, including the cerebellum, previously related to interoceptive accuracy. The anatomical changes were positively correlated with cognitive flexibility scores. Albeit the small sample size and preliminary nature of the findings, these results provide support for the hypothesized creativity-motor connection. The results are compared to other contemplative studies, and discussed in light of theoretical models integrating cognitive flexibility, embodiment and the motor system.

## Introduction

### Creativity, Training, and Health

The lexeme in the English word *creativity* comes from the Latin term creō, meaning “to create, make.” Thus, creativity means bringing into being, as it involves generation of novelty and transformation of existent information (Chávez-Eakle et al., 2007). Creativity requires, as well as generates, new information that transcends informational boundaries, yet is integrated with existing information in a manner exhibiting value (Horan, 2007). Behaviorally, the first component, namely the generation of new information, can be measured by divergent thinking tests; whereas the second component, concerning existing information constraints, can be measured by convergent thinking tests. Here we focus on divergent thinking, studying *ideational fluency* and *cognitive flexibility*, two important measures of creativity. Specifically, cognitive flexibility is diminished in several neurodegenerative conditions, such as Parkinson’s (Tomer et al., 2002). Thus, sustaining and improving cognitive flexibility may serve a significant role in maintaining cognitive and emotional well-being and health (Schmid, 2005). In the current study, we aimed at investigating the link between flexibility of behavior and cognitive flexibility. To this end, we employ *Quadrato Motor Training* (QMT by Patrizio Paoletti – see below), which requires constant flexibility in the movement and behavior, and assess its impact on cognitive flexibility and ideational fluency using the AUs Task (Chermahini and Hommel, 2010).

**FIGURE 1:**
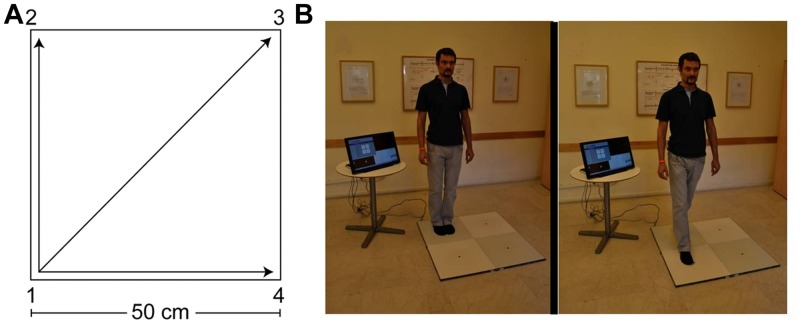
**The Quadrato Motor Training (QMT). (A)** A graphical illustration of the QMT. **(B)** A participant during the QMT while waiting for the next instruction (left) and following the instruction (right).

The QMT is a whole-body movement meditation, aimed at improving well-being, by enhancing attention, coordination and cognitive flexibility (Ben-Soussan et al., 2013, 2015). The QMT requires a state of enhanced attention, as it combines dividing attention to the motor response and cognitive processing for producing the correct direction of movement to the next point in the Quadrato space (Ben-Soussan et al., 2013; see **Figure 1**). QMT can further be viewed as ‘*Mindful movement,’* due to the increased awareness it requires to the body and its location in space. *Mindful movement* is a general term for practices that involve bringing awareness to the detailed experience of movement (Kabat-Zinn, 2009), such as walking meditation, Yoga and Tai Chi. Similar to other Mindful movement practices, QMT requires balance control, which is known to integrate inputs from the motor cortex, cerebellum, and basal ganglia, as well as feedback from vestibular and proprioceptive systems required to maintain an upright posture (Chang et al., 2010, 2014; Wayne et al., 2014). In line with that, a previous study demonstrated that a month of daily QMT practice significantly enhanced cerebellar oscillatory function (Ben-Soussan et al., 2014a). Yet, in comparison to other Mindful movement practices, QMT has the advantage of being a relatively short training (possibly several minutes), and can be practiced in limited spaces. These unique aspects render the QMT a technique warranting scientific exploration, with the future aim of implementing this technique in various health-promoting and educational setups.

We have previously reported that a single session of QMT increased cognitive flexibility, but not fluency, as well as improved spatial cognition and reflectivity (Ben-Soussan et al., 2013, 2014b) in contrast to two different control groups controlling for cognitive and motor load.

Long-term effects of a month of daily QMT on divergent thinking were previously studied in comparison to simple walking training (WT; Venditti et al., 2014). Both fluency and flexibility increased only in the QMT group (Venditti et al., 2014). These significant differences might have stemmed from the cognitive aspect of the contemplative movement (QMT), relatively missing from the non-contemplative simple walking (WT).

This has led us in the present study (Study A) to compare 4-weeks of daily QMT to two control groups, tapping on the cognitive and motor aspects of the QMT: Verbal Training (VT, identical cognitive training with verbal response) and Simple Motor Training (SMT, similar motor training with reduced choice requirements). At the same time, the present study replicates the 1-month QMT in a different language. In this study, we predicted that: (1) cognitive flexibility would increase following QMT compared to the other control groups, while (2) fluency is predicted to increase in both the QMT and the VT groups.

A direct continuation of Study A is to start uncovering the possible underlying mechanism mediating QMT-induced enhancement in divergent thinking. Indeed, a fascinating and under-explored aspect of creativity is its possible connection to the motor system, suggested by several authors (Cotterill, 2001; Dietrich, 2004; Chávez-Eakle et al., 2007; Vandervert et al., 2007; Carruthers, 2011; Koziol et al., 2012). Building on the previously hypothesized creativity-motor connection, we set out in the pilot Study B to examine a possible correlation between change in divergent thinking measures and structural changes in brain regions related to motor activation. To this end, we employed structural Magnetic Resonance Imaging (sMRI), and investigated structural changes and AU scores following 4-weeks of daily QMT. Here, we predicted that: (1) QMT would induce anatomical changes in motor regions; and (2) anatomical changes in motor regions would be correlated with increased cognitive flexibility.

## Study A

### Methods

#### Participants and Design

Twenty-seven female students (mean ± SD age: 24 ± 3 years) participated in the study, none of whom practiced QMT before. All were right-handed with no medical history that might affect their performance. Since gender-dependent differences have been frequently observed in both the motor and the cognitive realms (Baron-Cohen and Hammer, 1997) we chose to focus here on females. The study was conducted in the EEG/MEG unit at the Gonda Brain Research Center, and was approved by the ethics committee of Bar-Ilan University.

Upon entering the lab, the participant signed a written informed consent. Subsequently, participants were seated in a quiet room, in front of a computer screen and completed the AU Task. All data were collected both before and after 4 weeks of daily practice. Participants were randomly allocated to one of three groups: (1) Quadrato Motor Training (QMT- three choices and whole-body response); (2) SMT (one choice and whole-body response); and (3) VT (three choices and verbal response). Although the initial group sizes were identical (*n* = 9 each), the final number of participant finishing the 4-weeks training varied between the groups (QMT, *n* = 6; SMT, *n* = 7; and VT, *n* = 5).

#### Training Groups

##### Quadrato Motor Training

The QMT, by Patrizio Paoletti, requires standing at one corner of a 0.5 m×0.5 m square and making movements in response to verbal instructions given by an audio tape recording. There were three optional directions of movement. The instructions direct participants to keep the eyes focused straight ahead, hands loose at the side of the body. They are also told to immediately continue with the next instruction and not to stop due to mistakes. At each corner, there are three possible directions to move (for example, from corner 1 the participant can move to corner 2, to corner 3 or to corner 4). The training thus consists of 12 possible movements (3 directions × 4 corners): 2 forward, 2 backward, 2 left, 2 right and 4 diagonals. The participant is required to move from one corner to another according to the number on the recording. For example, if the sequence required is 1, 2, 1, 2, 1, 2, 3, 2, 4, 3, 1…. this means moving to the first corner, then to the second, then back to the first, and so on (see **Figure 1**). The daily training consisted of a sequence of 69 commands, lasting 7 min. For additional data regarding the training groups, see Ben-Soussan et al. (2013).

##### Simple Motor Training

The SMT group moved from corner to corner on the square in exactly the same manner as the QMT group (pace, duration, auditory cue), but their movement was consistently 1-2-3-4-1 etc. This group also practiced with the same recordings as the QMT group. However, while the QMT group was told that each number represented a different corner of the square, the SMT group was told to simply begin at a certain corner and to continue to the next corner clockwise in response to the instructions. That is, regardless of the number specified on the tape, they always moved in the same sequence. This reduced the uncertainty regarding the direction of the movement, compared to the QMT group. The SMT group thus provided a control of similar motor performance but with reduced cognitive demands.

##### Verbal Training

The VT group was designed to control for the motor load while keeping the same cognitive load and uncertainty. The participants, who were instructed to only make verbal responses, stood 1 m in front of the square, but did not move on the corners at all. Instead, they responded to the taped commands verbally by stating what direction of movement would be required in order to reach the corner specified by the command. For example, for a movement from corner 1 to corner 2, they were required to say “straight.” All other training parameters were kept identical to the QMT (pace, duration, auditory cue).

#### The Alternate Uses (AUs) Task

The AU Task is an established measure assessing creativity (Guilford et al., 1978). Two main features of creativity are *fluency*, defined as the total number of ideas generated, and *flexibility*, defined as the tendency to generate a heterogeneous pool of responses, or to use a variety of categories and themes when producing ideas (Guilford, 1950; Guilford et al., 1978; Runco, 1986). Flexibility conveys information that is not conveyed by fluency (Guilford, 1968; Runco, 1986). This task was chosen here as it was previously used to assess change in cognitive flexibility following motor training (Netz et al., 2007; Venditti et al., 2014).

In a previous study with the aim of examining changes in creativity following training (reported in Ben-Soussan et al., 2013), we clustered a 908-word database developed by Levy-Drori and Henik (2006), using hierarchical cluster analysis, and grouped those words having similar ratings of concreteness, availability of context and familiarity assigned by Levy-Drori and Henik. Concreteness was rated by them on a scale from one to seven, where “1” indicated very low concreteness (very abstract word) to “7” which indicated a very concrete word. Availability of context was defined as ranging from 1 to 7, with “1” indicating that it is very difficult to think of a context and “7” indicating that it is very easy. Familiarity was defined as ranging from “1” indicating that it is not very familiar and “7” indicating that it is very familiar. We then chose 18 words from the three largest clusters for which the level of concreteness, similarity on familiarity and availability features were highest (see Supplementary Table S1). These 18 words were divided into two groups (nine words in each group). A total of 60 participants received one of the two lists, and were asked to produce as many AUs as possible, 1 min being allocated for each item. Each word was shown on a single page on which the participant had to write down the various uses. The scores for each word were analyzed by counting the number of AUs produced. The words were then divided into six sets of three words so that each set had a similar rating for familiarity, concreteness and availability. One of the sets (set d) resulted in higher fluency scores (see Supplementary data, where corrections maintain all reported results).

The presentation order of each set of words was counterbalanced using a Latin square (see Supplementary Table S2). Three names of objects were shown on a computer screen before the training and after the training (at the beginning and at the end of the month). Six pairs of sets (e.g., a–f) were used with internal order counterbalanced across six participants. The internal order of the three items per set (Table S1 in supplementary material) was presented in a random manner. Importantly, sets a, b, c, d, e, and f each appear once in each ordinal position.

In this task, the participant is required to name as many different ways in which a given item might be used, within a certain time frame (1 min). The fluency score was defined as the mean number of uses given by the participant for the three items. On the basis of all the uses made by the participants, 10 independent categories were defined across all the items. These included broad categories of usage such as “a weapon” or “a costume.” The flexibility score was defined as the mean number of different categories employed by the participant across all three words presented (Russ, 1998). Hence, in order to calculate the flexibility score, all responses for a given item were first divided into the different independent categories. Two independent judges who were naïve to the identity of the participants and their training groups scored the test independently for flexibility, and consistency between judges was tested. We examined the correlation between the scores of the two judges using a two-tailed Pearson correlation coefficient test. A high correlation was found between scores given by the two judges both in the scores before the training and after the training: *r* = 0.88 and 0.86, *n* = 18, respectively.

### Statistical Analysis

We ran a Group (QMT, SMT, VT) × Training (pre, post) analysis of variance (ANOVA) for creativity (separately on fluency and flexibility scores). Then, *post hoc* paired *t*-tests were conducted.

### Results

**FIGURE 2:**
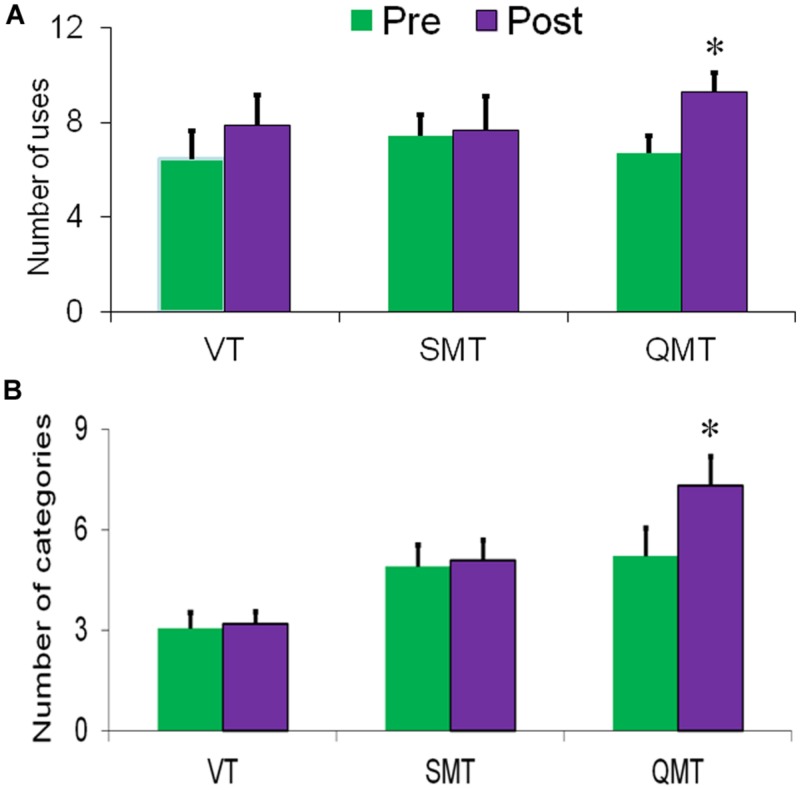
**Change in creativity as a function of Group and Training. (A)** Fluency and **(B)** Flexibility. Data are expressed as mean ± SEM, *^∗^p* < 0.05.

The first ANOVA, conducted for fluency, revealed a main effect for Training [*F*(1,15) = 8.80, MSE = 17.25, *p* = 0.01], with post-training being generally higher compared to pre-training (**Figure 2A**). Albeit the Group × Training interaction was not significant [*F*(2,15) < 1], we tested directly each group’s effect on fluency, to better evaluate the results compared to our previous studies. *Post hoc t*-tests showed that fluency significantly increased only in the QMT group [*t*(5) = -4.21, *p* < 0.01], and not in the SMT or VT groups [*t*(6) = -0.31, *t*(4) = -1.33, *ns*, respectively]. The second ANOVA conducted for flexibility similarly yielded a main effect for Training [*F*(1,15) = 8.01, MSE = 5.81, *p* < 0.05]. In addition, a significant Group × Training interaction was found [*F*(2,15) = 5.20, MSE = 0.727, *p* < 0.05]. For the QMT group, flexibility significantly increased [*t*(5) = -3.20, *p* < 0.05] in contrast to the SMT and VT groups who showed no change following training (see **Figure 2B**).

Importantly, correcting for AU set d did not change these results (see supplementary materials).

## Study B

### Methods

#### Participants and Design

Three healthy women participated in this pilot study (mean ± SD age: 41.5 ± 11.4 years), none of whom practiced QMT before, with no previous head injury which might have affected their brain structure. In this study, the QMT sequence consisted of 258 commands, and lasted 12 min. The reason for the change in procedure was due to the fact that this study is a part of a larger study conducted with the longer QMT practice paradigm aimed at examining longer sequences for neurodegenerative disease. The study took place at the St. Andrea Hospital, Rome. Upon entering the lab, the participant signed a written informed consent. sMRI was acquired immediately after the AU Task. The study was approved by the ethical committee of Università Campus Bio-Medico di Roma. The AUs task was employed similarly to the one reported in Study A, with different sets given to the participants before and following a month of QMT training. The AU task was completed prior to entering the magnet.

#### MRI Data

##### MRI Scans

For each subject, high-resolution 3D T1-weighted sMRI and diffusion tensor imaging (DTI) data were acquired at the beginning (pre-QMT) and after 4-weeks of daily QMT practice (post-QMT) using a Siemens MAGNETOM Sonata (Erlangen, Germany) 1.5 T scanner (sMRI: 3D T1-weighted MP-RAGE sequence, TR = 3000 ms, TE = 4.38 ms, flip angle = 15°, matrix = 192 × 192, FOV = 240 mm^2^, 160 sagittal slices, voxel size = 1.25 mm × 1.25 mm × 1.20 mm; DTI: 12 non-collinear direction sequence, TR = 7100 ms, TE = 94 ms, flip angle = 90°, matrix = 256 × 192, FOV = 240 mm × 320 mm, b = 0 and 900 s/mm^2^, 48 axial slices, voxel size = 1.25 mm × 1.25 mm × 3.0 mm). We computed two anatomical measures: gray matter (GM) volume, and fractional anisotropy (FA). GM volume is a measure of the amount of cortical GM corresponding to each region and is computed using both surface area and cortical thickness (Frye et al., 2010). FA is a marker of white matter integrity, which is thought to reflect anatomical features of white matter, such as axon caliber, fiber density and myelination (Scholz et al., 2009).

##### Image Analysis

The sMRI data were analyzed using the voxel-based morphometry technique (VBM, Good et al., 2001). DTI data were analyzed using the Tract-Based Spatial Statistics (TBSS, Smith et al., 2006); SMRI were longitudinally processed with VBM2 toolbox^1^ of SPM2^2^. In short, pre-QMT images were aligned to the T1 template and, then, post-QMT images were co-registered to the pre-QMT T1-aligned scans. All scans were bias corrected and segmented into gray and white, and CSF compartments. GM maps were normalized to MNI atlas space (1 mm × 1 mm × 1 mm voxels) and smoothed using an 8 mm FWHM Gaussian kernel. Preprocessing of DTI data was conducted with FSL^3^. DTIs were corrected for motion and eddy currents distortions and then skull-stripped using the Brain Extraction Tool (BET; Smith, 2002). Maps of FA were computed by fitting a tensor model to the raw diffusion data using the FMRIB’s Diffusion Toolbox (FDT). These maps were projected onto a mean FA tract skeleton, before applying voxelwise within-subject statistics to compare them between time points, as described elsewhere (Scholz et al., 2009).

### Statistical Analysis

Voxel-based morphometry technique data were analyzed using a general linear model (GLM). Anatomical localization was carried out with the MSU–MNI Space Utility toolbox of SPM using the AtlasQuery FSL tool. Statistical analysis of TBSS data was performed by using a permutation-based inference tool for non-parametric statistical thresholding. Pre- and post-QMT FA and VBM data were compared by using a paired *t*-test, with subject’s age as a covariate, controlling for the potential effect of this variable (Luders et al., 2009). Significance level for the *t*-tests was set with a minimum cluster size of 700 voxels for the GM volume (Brubaker et al., 2010) and one of 100 voxels or more for the FA as the cluster-size threshold (Ocklenburg et al., 2013).

### Results

#### Alternate Uses Task

**FIGURE 3:**
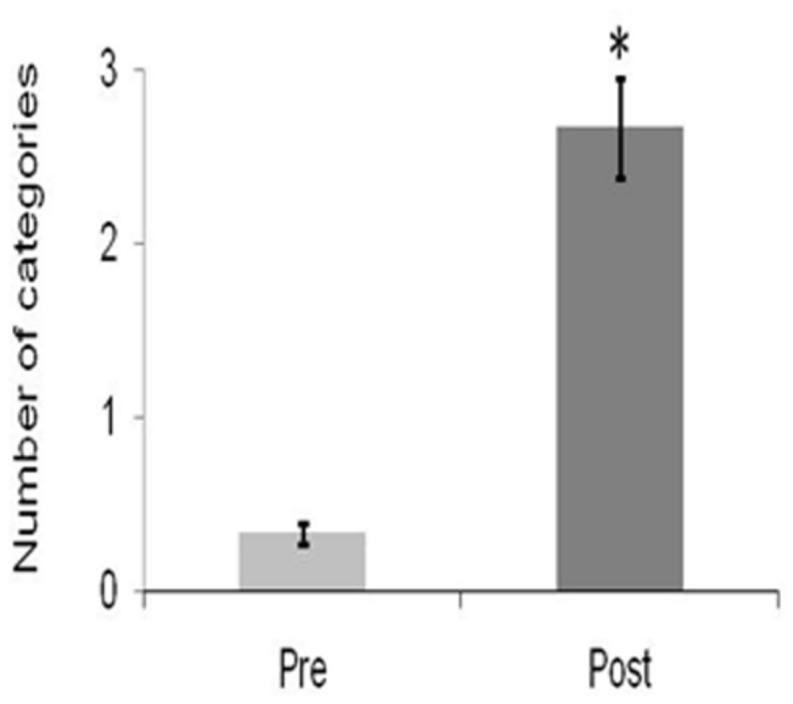
**Change in creativity as a function of Training for Flexibility.** Data are expressed as mean ± SEM, *^∗^p* < 0.05.

Similar to the results of Study A, flexibility significantly improved following 4-weeks of daily QMT [*t*(2) = -6.05, *p* < 0.05; **Figure 3**]. Although fluency also increased, it did not reach significance [*t*(2) = -0.39, *ns*].

#### Brain Anatomy Results

**FIGURE 4:**
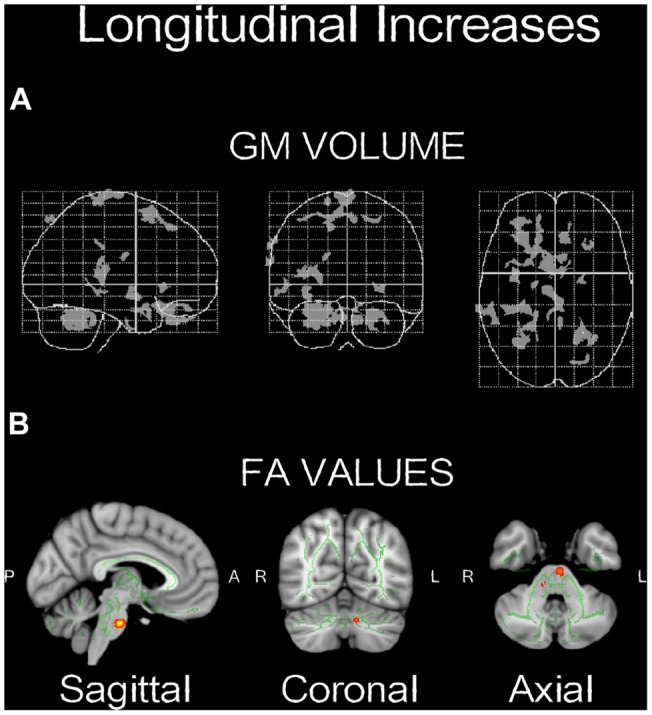
**Neuroplasticity following longitudinal (4-weeks) daily QMT. (A)** Significant increases in GM volume (*p* < 0.001, uncorrected) projected onto the glass brain; **(B)** Significant increases in FA values (*p* < 0.01, uncorrected) onto brain template. Red-yellow color scale is related to the *p*-values (in which yellow is highest).

Voxel-based morphometry technique analysis results showed significant (*p* < 0.001, uncorrected) GM volume increases, localized in left and right cerebellum, and frontal areas, mainly in the inferior frontal and middle frontal gyri (**Table 1**; **Figure 4A**). FA analysis results showed significant (*p* < 0.01, uncorrected) increases localized mainly in the middle cerebellar peduncles (**Table 2**; **Figure 4B**).

**Table 1:** Significant increases in GM volume after 4-weeks of daily QMT.

			Coordinates						
*k*	*p*-value	*Z*-value	*x*	*y*	*z*	Hemisphere	Lobe	Region	BA
1584	0.000	3.63	24	-52	-22	Right cerebellum	Anterior lobe	Culmen	
							Posterior lobe	Cerebellar tonsil	
								Declive	
								Tuber	
								Uvula	
1128	0.000	3.79	-6	13	65	Left cerebrum	Frontal lobe	Medial frontal gyrus	6
								Middle frontal gyrus	6
								Superior frontal gyrus	8
851	0.000	4.07	-15	21	-23	Left cerebrum	Frontal lobe	Inferior frontal gyrus	47
								Middle frontal gyrus	11
								Orbital gyrus	47
								Subcallosal gyrus	47
								Superior frontal gyrus	11
706	0.000	4.34	-17	-37	-29	Left cerebellum	Anterior lobe	Culmen	
580	0.000	4.32	-32	-62	-33	Left cerebellum	Posterior lobe	Cerebellar tonsil	
								Culmen	
								Declive	
								Pyramis	
								Tuber	
								Uvula	
							Anterior lobe	Culmen	
471	0.000	3.88	-37	-5	-7	Left cerebrum	Sub-lobar	Claustrum	
								Extra-nuclear	13
							Temporal lobe	Superior temporal gyrus	38
297	0.000	4.10	-54	-37	-3	Left cerebrum	Temporal lobe	Middle temporal gyrus	21
								Superior temporal gyrus	22
262	0.000	4.18	11	28	-33	Right cerebrum	Frontal lobe	Orbital gyrus	11
								Rectal gyrus	11
102	0.001	3.19	21	-6	-38	Right cerebrum	Limbic lobe	Parahippocampal gyrus	35
								Uncus	36

*Cluster extension (k), which represents the number of contiguous voxels passing the threshold of ≥100. All clusters meet the significance level set at *p* < 0.001 uncorrected. Neuroanatomical labels are referred to the Talairach coordinates *x, y, z*, by means of the MSU SPM tool.*

**Table 2:** Significant increases in FA values after 4-weeks of daily QMT.

		Coordinates			
*k*	*p*-value	*x*	*y*	*z*	*WM structures*
283	0.010	102	102	41	Middle cerebellar peduncle
59	0.004	94	107	38	Corticospinal tract L
31	0.008	100	62	40	Anterior thalamic radiation L
27	0.008	54	71	74	Posterior thalamic radiation R

*The number of voxels (k) represents the number of voxels belonging to certain WM structures. Significance level set at *p* < 0.01 uncorrected. Neuroanatomical localization was obtained using AtlasQuery FSL tool with JHU White-Matter Tractography and the JHU ICBM-DTI-81 White-Matter Labels as reference atlases.*

We then examined the correlation between the anatomical changes and changes in flexibility, both of which significantly increased post-training. Multiple regression analyses were performed to investigate whether GM and FA map changes were correlated with the change in flexibility, as previously reported for a similar group size (Ben-Soussan et al., 2015). We used the results of VBM and TBSS analyses as explicit masks to ensure that only the appropriate GM and fiber ROI which changed following the training were included in the analysis (Denier et al., 2013). These masks were subsequently inserted as explicit masks in two correlation analyses with flexibility, one for the GM and one for the FA.

**FIGURE 5:**
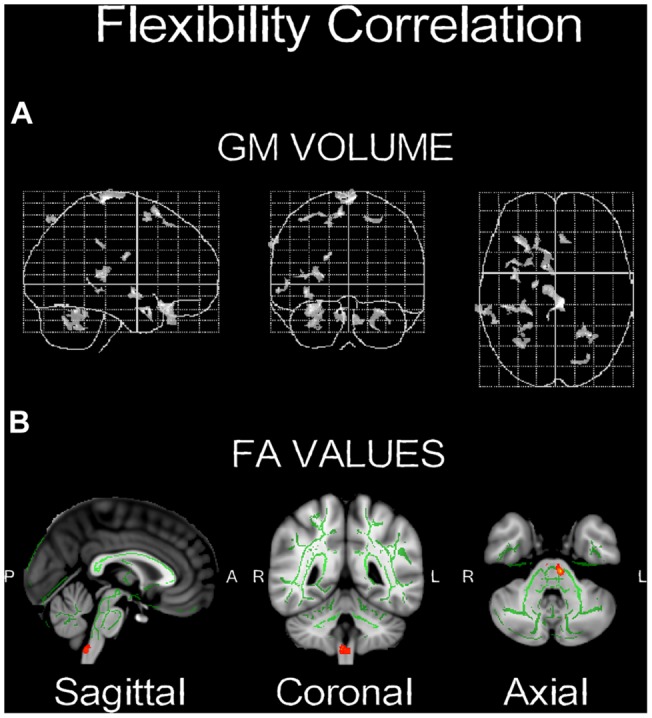
**Significant correlations between change and GM volume **(A,B)** FA values.** Red-yellow color bar range is based on min-max statistical threshold. Refer to corresponding supplementary tables for more neuroanatomical and statistical details (*n* = 3).

As seen in **Figure 5A**, a positive correlation (*p* < 0.005, *n* = 3) was found between change in flexibility and the GM increment in the right cerebellum and the superior frontal gyrus (**Table 3**). In addition, a positive correlation (*p* < 0.05) was found between increased flexibility and FA changes, mostly located in the left corticospinal tract and the middle cerebellar peduncles (**Table 4**; **Figure 5B**).

**Table 3:** Positive correlation between flexibility values and GM maps showing volume increases after 4-weeks of daily QMT.

			Coordinates						
*k*	*p*-value	*Z*-value	*x*	*y*	*z*	Hemisphere	Lobe	Region	BA
612	0.000	3.73	21	-56	-37	Right cerebellum	Anterior lobe	Culmen	
							Posterior lobe	Cerebellar tonsil	
								Declive	
301	0.001	4.41	-6	18	62	Left cerebrum	Frontal lobe	Superior frontal gyrus	6, 8
292	0.001	3.47	-29	-64	-32	Left cerebellum	Posterior lobe	Cerebellar tonsil	
								Pyramis	
								Tuber	
								Uvula	
								Culmen	
								Declive	
							Anterior lobe	Culmen	
248	0.002	3.83	-37	-2	-7	Left cerebrum	Sub-lobar	Extra-nuclear	13
								Claustrum	
235	0.000	3.76	-25	22	-18	Left cerebrum	Frontal lobe	Inferior frontal gyrus	47
								Middle frontal gyrus	11
180	0.005	3.55	-37	9	58	Left cerebrum	Frontal lobe	Middle frontal gyrus	6
								Superior frontal gyrus	6
133	0.002	3.83	-37	-4	-5	Left cerebrum	Temporal lobe	Middle temporal gyrus	21
								Superior temporal gyrus	22
65	0.001	3.35	5	31	-28	Right cerebrum	Frontal lobe	Orbital gyrus	11
								Rectal gyrus	11

*k ≥ 100; *p* < 0.005 uncorrected. Refer to **Table 1** for a detailed explanation of the table layout.*

**Table 4:** Positive correlation between flexibility values and FA maps showing increases after 4-weeks of daily QMT.

		Coordinates			
*k*	*p*-value	*x*	*y*	*z*	WM structures
21	0.018	96	101	37	Corticospinal tract L
5	0.047	102	101	41	Middle cerebellar peduncle

*Significance level set at *p* < 0.05 uncorrected. Refer to **Table 2** for a detailed explanation of the table layout.*

## Discussion

### QMT Improves Divergent Thinking Creativity

The results of Study A show that 4-weeks of daily QMT practice induce increased cognitive flexibility and ideational fluency, which was not the case in either the SMT group or in the VT group, representing the motor and cognitive aspects of the training, respectively (**Figure 2**). This is in line with our first hypothesis, and with two previous studies. In the first relevant study, we reported findings regarding the effects of a single session of QMT (Ben-Soussan et al., 2013), where a similar increase in flexibility was observed in contrast to SMT or VT. However, no significant change was reported for fluency. Our results show that the increase in flexibility was not significantly different between one session and 4-weeks of training, possibly suggesting that participants might have reached a ceiling effect in the AU task after one session, underscoring the need to utilize in the future different tasks to assess QMT-induced cognitive flexibility. Yet, as opposed to one session of training (Ben-Soussan et al., 2013), long-term QMT also increased ideational fluency, which is probably mediated by an accumulated effect of daily QMT sessions. Yet, the fluency results should be treated with caution as the Group × Training interaction was not significant. We further acknowledge the reduction in statistical power given participant attrition, in part because participants who successfully complete all follow-up measurements may have differed from those respondents lost to attrition. Nevertheless, no baseline differences were found in the AU scores between those who continued and those who dropped out (see Supplementary data and Table S4).

In another recent study, we examined the effects of a month of daily QMT as opposed to simple WT, tapping the motor aspect of QMT (Venditti et al., 2014). This study reported increased flexibility and fluency following QMT but not WT. Our results replicate the previous results in the QMT group, as expected. This demonstrates that there is no language bias affecting the results, as the current study was conducted with Hebrew speakers, whereas the previous study was conducted with Italian speakers. Importantly, while we hypothesized an increase in fluency following VT, tapping the cognitive aspect of the QMT, no change in fluency occurred in both the VT and SMT groups. This suggests that the combination of the cognitive and motor aspects of the QMT, and not the cognitive aspect *per se*, renders the fluency enhancement. Put differently, we suggest that this stems from the complexity of the movement, indicating that it is the combination of the motor and cognitive aspects embedded in QMT, which is important for increasing fluency.

In Study B, flexibility again significantly increased, as expected (**Figure 3**). Yet, although a trend was observed for fluency, it did not reach significance, possibly due to the small sample size. However, the possibility that the discrepancy between the results of Study A and Study B in terms of fluency is due to the altered QMT sequence in the second study (12 min in Study B vs. 7 min in Study A) cannot be ruled out. Therefore, the effect of QMT sequence length on different aspects of creativity should be further examined, underlying the importance of examining additional sequences of the QMT.

### QMT as a Mindful Practice

Our results are in line with the proposal that contemplative practices increase creativity (Horan, 2007), albeit early evidence is inconsistent with that, possibly due to the wide variety of meditation techniques and creativity measures employed (Horan, 2007; Lippelt et al., 2014). Although there are many meditative techniques, we adopt here the conceptualization of Lutz et al. (2008), grossly dividing meditation into two forms: focused attention – learned control over the focus of one’s attention by using a stable object with the goal of quieting the mind, and open monitoring (OM) – maximizing the breadth and clarity of maintained attention in order to bring higher momentary awareness to internal processes (the latter includes for example Mindfulness and Zen). Importantly, when focusing only on studies investigating the effect of OM techniques on creativity, the overall picture is of OM practices increasing divergent thinking (Cowger and Torrance, 1982; Zabelina et al., 2011; Colzato et al., 2012; Greenberg et al., 2012; Ding et al., 2014).

Here, we wish to draw attention to the mindful nature of QMT, supported by the similarity of the training effects in enhancing divergent thinking. Mindful practices have been noted in the literature using varied terminology depending on the discipline called upon, including “the act of becoming more aware,” “a reflective act,” or “mindfulness” (Depraz et al., 2000). It has been claimed that the mindful act has three interdependent phases: a first phase of suspension from the habitual act of allowing the mind and body to go where they want; a second phase of redirection of attention inwardly; and a third phase of receptivity toward the experience (Depraz et al., 2000). Mindfulness meditation, as conceptualized in the Western tradition, incorporates all three phases (Kabat-Zinn, 2009). Similarly, QMT can be thought of as training in all the above three phases. First, one suspends the automatic movement. Then, one redirects attention toward the external cue and the internally generated movement. Finally, one quietly stands in a receptive manner in between instructions, without correcting motor or decision errors. QMT is also dependent on the moment by-moment re-investment of attention. In support of that, we reported increased ability to respond in a non-habitual fashion following 5–12 min QMT (Moore and Malinowski, 2009; Ben-Soussan et al., 2014b). To sum, QMT can be considered to be a Mindful Movement practice, and similarly to other OM practices, leads to increased divergent thinking.

### Structural Changes Following QMT

Structural changes in GM volume were found mainly in the left and right cerebellum, as well as in the left frontal lobe, whereas the main FA increase was found in the middle cerebellar peduncle (**Figure 4**). The changes in the cerebellum and the frontal cortex could be explained by their role in the acquisition of motor sequences (Exner et al., 2002). The cerebellum is important for the integration of somatosensory and motor information relevant to coordinate context-dependent planning and execution of coordinate motion (Ivry, 2000; Tesche and Karhu, 2000). Although interoception was not directly measured here, it is important to note in the context of this special issue, that the cerebellum is closely linked to interoceptive awareness reflecting explicit awareness of bodily processes (Critchley et al., 2004; Mercader et al., 2010). The left frontal regions, mainly in the inferior frontal and middle frontal gyri, are related to motor learning, action observation and intention understanding (Exner et al., 2002; Dapretto et al., 2005). In addition, both the inferior frontal and the middle frontal gyri (see **Table 1**) have been consistently related to working memory and response inhibition (Leung et al., 2002; Morin and Michaud, 2007; Swick et al., 2008) and selection among competing alternatives (Paulesu et al., 1993) which may increase following QMT. Notably, the spatial proximity of the GM and FA increases in the cerebellum following the training suggests that the QMT-induced increase in GM volume is related to an altered organization of underlying white matter pathways. Importantly, we have previously reported that following 3-months QMT cerebellar GM volume and FA increased, in positive correlation with increased brain-derived neurotrophic factor (BDNF) level (Ben-Soussan et al., 2015), a neurotrophin closely linked to interoceptive awareness (Mercader et al., 2010). In addition, QMT was previously reported to increase cerebellar low-rhythm activity (Ben-Soussan et al., 2014a). This has led to a theoretical account emphasizing the cerebellum as an important candidate which possibly mediates the QMT effects on well-being (Ben-Soussan et al., 2015). The current anatomical results provide further support to such a proposition, and show that 1-month QMT is sufficient to induce measurable cerebellar anatomical alterations.

The current results are in line with previous meditation studies which have found activation in areas closely linked to motor learning, such as the frontal gyrus and the cerebellum. For example, Pagnoni and Cekic (2007) reported increased GM volume in Zen meditation practitioners compared to matched controls, especially in the putamen which is closely involved in the control of voluntary movement (Nambu et al., 2002). This result was interpreted as being related to the cognitive processes engaged by meditation, and especially to the conscious regulation of attention and posture (Pagnoni and Cekic (2007). Vestergaard-Poulsen et al. (2009) examined the effects of Tibetan Buddhist meditation, and found that in addition to increased GM density in the medulla oblongata, left superior and inferior frontal gyri, and left fusiform gyrus, an increment was found in the anterior lobe of the cerebellum in the meditator group compared to the control group. In addition, Hölzel et al. (2011) studied the effect of mindfulness-based stress reduction (MBSR) in meditation-naïve participants, before and after 8-weeks of training. In addition to other regions, they found increased GM density in the cerebellum in the MBSR group compared to controls. Hölzel et al. (2011) address their cerebellar-related results in relation to its role in the regulation of emotion and cognition. They further mention the claim made by Schmahmann (2004), that in the same way that the cerebellum regulates the rate, force, rhythm, and accuracy of movements, it also regulates the speed, capacity, consistency, and appropriateness of cognitive and emotional processes.

Compared to sitting meditation, the anatomical examination of whole-body movement-based contemplative practices is surprisingly rare. One exception is a recent study examining the effects of Tai Chi Chuan (TCC), a whole-body contemplative practice involving movement, such as weight-shifting between the right and left legs and asymmetrical diagonal leg movements (Wei et al., 2013). Wei et al. (2013) demonstrated that TCC practitioners showed greater cortical thickness in prefrontal cortex and temporal cortex relative to the matched control group. Interestingly, neuroimaging studies have consistently shown similar findings following aerobic exercise, as this was shown to lead to increased gray and white matter volume in the prefrontal cortex of older adults (Colcombe et al., 2006). In addition, greater amounts of physical activity, such as walking, are associated with sparing of prefrontal and temporal brain regions of late adulthood (Erickson et al., 2010). Taken together, the above studies show that both sitting and movement contemplative practices induce neuroplasticity in motor regions. This strengthens the claim that the results shown here might not be due to the movement *per se*, but involve higher cognitive modulation.

### Creativity and the Motor System

Albeit the small sample size and the preliminary nature of the findings, a positive correlation was found between increased flexibility and cerebellar changes, both in the GM volume and the FA values (**Figure 5**). Due to the low power of the correlation analysis, this finding should be treated as being suggestive, but can still guide the hypotheses of future larger studies. This correlation supports the previously suggested link between creativity and the motor system (Dietrich, 2004; Carruthers, 2011; Koziol et al., 2012). For example, Cotterill (2001) suggested that: “If cognition is linked to overt or covert movement, intelligence becomes the ability to consolidate individual motor elements into more complex patterns, and creativity is the outcome of a race-to-threshold process which centers on the motor areas.” (p. 1). Similarly, Carruthers (2011) wrote: “creativity lies in the assembly and activation of action-schemata, with creative thoughts arising subsequently from the mental rehearsal of those actions” (p. 437). The creativity-motor connection has been advanced to the point that it has been provocatively claimed that: “we were not born to think. We were born to move. Human creative ideas are nothing in the absence of the manual dexterity that allows tools to be made, complex architecture to be constructed, art to be created, and instruments to be played” (Koziol et al., 2012, p. 515).

The motor-creativity hypothesized connection was previously supported by several reports. For example, a positive correlation was found between figural and verbal creativity and cerebral blood flow in the right cerebellum (Chávez-Eakle et al., 2007). Takeuchi et al. (2010) measured GM volume using voxel-based morphometry and found a positive correlation between divergent thinking and the right dorsolateral prefrontal cortex and the bilateral striata. In accord with our anatomical results, other reports have also emphasized the involvement of the cerebellum and the precentral gyrus in creative processes (Cotterill, 2001; Chávez-Eakle et al., 2007; Vandervert et al., 2007). The current findings also provide preliminary insight regarding the possible relationship between anatomical changes in motor-related areas and creativity.

## Conflict of Interest Statement

The authors declare that the research was conducted in the absence of any commercial or financial relationships that could be construed as a potential conflict of interest.

## Footnotes

1 http://dbm.neuro.uni-jena.de/vbm/

2 http://www.fil.ion.ucl.ac.uk/spm/

3 http://www.fmrib.ox.ac.uk/fsl
