# Supplementary material for: Embodied cognitive flexibility and neuroplasticity following Quadrato Motor Training
Source: Front Psychol. 2015 Jul 22;6:1021. doi: 10.3389/fpsyg.2015.01021 (PMC4511076; doi:10.3389/fpsyg.2015.01021)
Supplement: Supplementary file 1 [file Data_Sheet_1.DOCX]

**Supplementary data**

The original sets of AU task and the original presentation order are presented in Table S1 and S2. The sets of AU task before and following a month of training (AUpre and AUpost respectively) are reported in Table S3, including details of the frequency among the participants who remained in the study and those who dropped out. The frequencies of AU sets for each group in the study, before and after a month of training, including the expected and actual mean group fluency, are reported in Table S4. No baseline differences were found in the AU scores between those who continued and those who dropped out (*t*(24) = 0.62, - 1.24, *ns*, for fluency and flexibility, respectively).

In analyzing the data presented in Table S1, by means of one-way ANOVAs, we found no significant difference between these six sets (a-f) of the AU task in terms of word availability [F (5, 17) = .70, *ns*], familiarity [*F* (5, 17) = .51, ns] and concreteness [*F* (5, 17) = 1.78, *ns*]. However, there was a significant difference between the six sets in terms of fluency [*F* (5, 17) = 4.01, *MSE* = 2.67, p < .05], stemming from a higher number of uses in set d. Indeed, when excluding set d from the analysis, there was no significant difference between the other five sets [*F* (4, 14) = 2.47, *ns*]. However, since we used and report all six sets in this study (see Table S2 for set frequency in the different groups), we show here in all relevant cases that the results remain essentially the same when correcting the potential bias created by using set d. In order to test if the significant group difference stems from the completion of set d by several participants, we ran a Group (QMT, SMT, VT) x Training (pre, post) analysis of variance (ANOVA) separately for fluency and flexibility scores excluding these participants (3 QMT, 1 SMT and 1 VT participant). For Fluency, as before, after this correction the main effect for Training remained marginally significant [*F* (1, 2) = 4.51, *p* = 0.063], while the interaction was not significant [*F* (2, 9) = 1.32, *MSE* = 2.51, *ns*]. Importantly, for flexibility the Group x Training interaction remained significant [*F* (2, 9) = 8.23, *MSE* = 8.23, *p* < .01].

Post-hoc t-tests revealed that both fluency and flexibility significantly increased following QMT (t(2) = -11, -5.05, p < 0.05, respectively), and not following SMT (t(5) = -0.12, -1.47, ns, respectively) or VT (t(2) = 0.97, 0.31, ns, respectively) for fluency and flexibility, respectively.

We also introduced another way of correction correcting the fluency results, without excluding participants. In this case, we corrected the fluency results of the participants who completed set d by a factor: the mean fluency of the AU sets excluding set d is 5.088, while set d scores 7, thus the correction factor is 0.726 (=5.088/7). An ANOVA on the corrected fluency values yielded a marginally significant Training effect [F (1, 15) = 3.89, p = 0.067]. Post-hoc t-tests revealed that fluency significantly increased following QMT (t(5) = - 4.05, p < 0.01), and not following SMT (t(6) = 0.18, or VT (t(4) = - 0.62, ns), for fluency and flexibility, respectively.

Table S1. **The pilot results for the Alternative Uses (AU) task**. The pilot results for the AU task versions with the concreteness, availability and fluency (*n* = 60).

| **AU version** | **Word** | **Availability** | **Familiarity** | **Concreteness** | **Fluency** | | **Set Fluency** | |
| --- | --- | --- | --- | --- | --- | --- | --- | --- |
|  |  | **Mean** | **Mean** | **Mean** | **Mean** | **SD** | **Mean** | **SD** |
| **a** | Boot | 4.67 | 4.22 | 6.56 | 4.17 | 1.86 | 5.10 | 2.29 |
|  | Envelope | 4.70 | 4.97 | 6.37 | 5.14 | 1.96 |  |  |
|  | Bench | 4.85 | 4.43 | 5.83 | 6.00 | 3.07 |  |  |
| **b** | Screwdriver | 4.60 | 3.86 | 6.09 | 4.83 | 0.90 | 5.00 | 1.27 |
|  | Ladder | 5.00 | 4.37 | 6.03 | 5.50 | 0.96 |  |  |
|  | Drawer | 4.54 | 4.29 | 6.29 | 4.67 | 1.97 |  |  |
| **c** | Stand | 4.83 | 4.17 | 5.94 | 3.67 | 2.49 | 4.16 | 2.03 |
|  | Antenna | 4.40 | 4.41 | 6.33 | 3.50 | 1.26 |  |  |
|  | Tie | 4.50 | 4.28 | 6.33 | 5.33 | 2.36 |  |  |
| **d** | Shutter | 4.65 | 4.66 | 6.37 | 5.50 | 3.57 | 7.00 | 3.40 |
|  | Flowerpot | 4.83 | 4.37 | 6.40 | 8.00 | 2.24 |  |  |
|  | Box | 4.50 | 4.22 | 6.30 | 7.50 | 4.39 |  |  |
| **e** | Bowl | 4.73 | 4.37 | 6.20 | 5.20 | 1.17 | 5.62 | 2.58 |
|  | Umbrella | 5.41 | 4.23 | 6.49 | 5.67 | 3.30 |  |  |
|  | Scarf | 4.43 | 4.13 | 6.52 | 6.00 | 3.27 |  |  |
| **f** | Vase | 4.43 | 3.97 | 6.56 | 5.50 | 2.06 | 5.66 | 1.77 |
|  | handkerchief | 4.37 | 4.10 | 6.63 | 5.75 | 1.79 |  |  |
|  | Pillow | 4.67 | 4.97 | 6.52 | 5.75 | 1.48 |  |  |

Table S2. **The original presentation order of the sets**. The original presentation order of the sets counterbalanced following a Latin square.

| Participant | Session 1 | | Session 2 | |
| --- | --- | --- | --- | --- |
|  | before | after | before | after |
| 1 | a | b | f | e |
| 2 | b | c | a | f |
| 3 | c | d | b | a |
| 4 | d | e | c | b |
| 5 | e | f | d | c |
| 6 | f | a | e | d |

Table S3. **Sets of AU task used in the study.** Sets of AU task before training at the beginning of the month and following a month of training (AUpre and AUpost respectively). The table further indicates the participants who remained in the study and those who dropped out.

| Group | AUpre | AUpost | Frequency  after attrition | Attrition |
| --- | --- | --- | --- | --- |
| QMT | a | f | 1 | 1 |
|  | b | a | 1 | 1 |
|  | c | b | 1 |  |
|  | d | c | 2 |  |
|  | e | d | 1 |  |
|  | f | e |  | 1 |
| SMT | a | f | 1 | 1 |
|  | b | a | 1 | 1 |
|  | c | b | 2 |  |
|  | d | c |  |  |
|  | e | d | 1 |  |
|  | f | e | 2 |  |
| VT | a | f |  | 2 |
|  | b | a | 1 | 1 |
|  | c | b | 2 |  |
|  | d | c | 1 |  |
|  | e | d | 1 |  |
|  | f | e |  | 1 |

Table S4. **Final AU sets’ frequencies.** AU sets’ frequencies for each group in the study, before and after a month of training, including the expected and actual mean group fluency.

|  | Pre | | | | | | | Post | | | | | | |
| --- | --- | --- | --- | --- | --- | --- | --- | --- | --- | --- | --- | --- | --- | --- |
| Group | AU set | Frequency | Pilot mean fluency | Expected mean fluency | | Actual mean fluency | | AU set | Frequency | Pilot mean fluency | Expected mean fluency | | Actual mean fluency | |
|  |  |  |  | Mean | SD | Mean | SD |  |  |  | Mean | SD | Mean | SD |
|  | d | 2 | 7 | 5.64 | 1.14 | 6.72 | 1.9 | c | 2 | 4.16 | 5.18 | 1.06 | 9.28 | 2.2 |
|  | e | 1 | 5.62 |  |  |  |  | d | 1 | 7 |  |  |  |  |
| QMT | b | 1 | 5 |  |  |  |  | a | 1 | 5.1 |  |  |  |  |
|  | c | 1 | 4.16 |  |  |  |  | b | 1 | 5 |  |  |  |  |
|  | a | 1 | 5.1 |  |  |  |  | f | 1 | 5.66 |  |  |  |  |
|  | c | 2 | 4.16 | 5.05 | 0.66 | 7.43 | 2.4 | b | 2 | 5 | 5.57 | 0.69 | 7.67 | 3.9 |
|  | e | 1 | 5.62 |  |  |  |  | d | 1 | 7 |  |  |  |  |
| SMT | f | 2 | 5.66 |  |  |  |  | e | 2 | 5.62 |  |  |  |  |
|  | a | 1 | 5.1 |  |  |  |  | f | 1 | 5.66 |  |  |  |  |
|  | b | 1 | 5 |  |  |  |  | a | 1 | 5.1 |  |  |  |  |
|  | c | 2 | 4.16 | 5.18 | 1.18 | 6.47 | 3.2 | b | 2 | 5 | 5.25 | 1.04 | 7.87 | 3.6 |
|  | e | 1 | 5.62 |  |  |  |  | d | 1 | 7 |  |  |  |  |
| VT | d | 1 | 7 |  |  |  |  | c | 1 | 4.16 |  |  |  |  |
|  | b | 1 | 5 |  |  |  |  | a | 1 | 5.1 |  |  |  |  |
